# Forms, Mechanisms, and Roles of Iconicity in Spoken Language: A Review

**DOI:** 10.1177/00332941241310119

**Published:** 2024-12-20

**Authors:** Harry Barker, Mirjana Bozic

**Affiliations:** Department of Psychology, 98528 University of Cambridge, Cambridge, UK

**Keywords:** Iconicity, sound symbolism, cross-modal correspondence, language evolution, neurobiology of language

## Abstract

Historically, debates over relationships between spoken lexical form and meaning have been dominated by views of arbitrariness. However more recent research revealed a different perspective, in which non-arbitrary mappings play an important role in the makeup of a lexicon. It is now clear that phoneme-sound symbolism - along with other types of form-to-meaning mappings - contributes to non-arbitrariness (iconicity) of spoken words, which is present in many forms and degrees in different languages. Attempts have been made to provide a mechanistic explanation of the phenomenon, and these theories largely centre around cross-modal correspondences. We build on these views to explore iconicity within the evolutionary context and the neurobiological framework for human language processing. We argue that the multimodal bihemsipheric communicative system, to which iconicity is integral, has important phylogenetic and ontogenetic advantages, facilitating language learning, comprehension, and processing. Despite its numerous advantages however, iconicity must compete with arbitrariness, forcing language systems to balance the competing needs of perceptual grounding of the linguistic form and ensuring an effective signal. We conclude that, on balance, iconicity should be viewed as integral to language, and not merely a marginal phenomenon.

## Introduction

The nature of the relationship between word forms and their meanings has long been a prominent question. Do words sound like what they describe, or is this relationship arbitrary? Modern linguistic theory has largely adopted the view that the phonological form of the lexical sign bears no resemblance to its meaning; the connection is arbitrary. This view is often attributed to Saussure (1959) who argued “Because the sign is arbitrary, it follows no law other than that of tradition, and because it is based on tradition, it is arbitrary”. However, the idea of arbitrariness was established much earlier by Locke (1690), who proposed that the existence of multiple languages is sufficient evidence to disprove a so-called ‘natural’ connection: if the properties of the concept or object that the word refers to are sufficient to determine a phonological form, then there should only be one language. The notion of arbitrariness provides a robust explanation for the origin of most words; for example, there is nothing about the phonological form of the word ‘pen’ to suggest that it should denote ‘an instrument for writing or drawing with ink’. It has simply been established that there is a conventionalised system of symbols shared by communities of users and passed from generation to generation - “there is no reason for you to call a dog ‘dog’ rather than ‘cat’ except for the fact that everyone else is doing it” (Pinker & Bloom, 1990, p. 728). Notable exceptions to the conventional approach, such as onomatopoeia, have been regarded as marginal phenomena within an arbitrary linguistic system, with Saussure (1959) arguing that “such words are never organic elements” of language. In addition, although onomatopoeic words seem to represent the sounds they symbolise, they too show evidence of arbitrariness. For example, the English expression for the sound of a rooster (*cock-a-doodle-doo*) differs from German and French expressions (*kikeriki* and *cocorico*, respectively), all of which are acoustically different from the actual sound made by a rooster (Perniss et al., 2010). There is therefore plenty of evidence to support the idea of conventionalised, wholly arbitrary mapping between phonological form and meaning in our communication system. Indeed, the idea of language as arbitrary dominates many of the prominent theories of language acquisition, production, and comprehension; where relatively little attention has been focused on determining how phonological and semantic representations might overlap.

However, in looking for an accurate account of non-arbitrariness in language, a broad-cross linguistic perspective is required, and more recent work has looked beyond Indo-European languages as well as at both signed and spoken languages (Perlman et al., 2018). This research has suggested a more textured view that non-arbitrariness may have a more important role in language than previously thought, and may in fact complement arbitrariness by conveying different linguistic advantages.

### Defining Forms of Non-Arbitrariness

When considering forms of non-arbitrariness in language, there has been much confusion over terminology despite attempts to clarify these uncertainties (e.g., Lockwood & Dingemanse, 2015; Sidhu, 2019). The term *iconicity* is usually taken to refer to a form-meaning resemblance that may apply to spoken, manual and written communication and is not limited to human language. In fact, there is evidence for iconicity in the communicative acts of non-human primates, which we will later explore in the context of human language evolution. *Sound symbolism,* which the current paper focuses on, is used to specifically refer to iconicity in spoken language, though these terms have previously been used interchangeably in much of the literature.

It has been argued that iconicity is not a binary property and may be present in different degrees, with a distinction between ‘absolute’ (imagic) and ‘relative’ (diagrammatic) iconicity (Dingemanse et al., 2015; Peirce, 1974); and ‘direct’ and ‘indirect’ iconicity (Masuda, 2007). Absolute iconicity is where there is a one-to-one mapping between phonological form and meaning. This may be ‘direct’ as in onomatopoeia, for example the sound of water leaking ‘*drip drop’* (English) or ‘*plitsch platsch’* (German), or ‘indirect’, such as the association between the phoneme /i/ and smallness in words like ‘*petite’* and ‘*tiny’*. Absolute iconicity in spoken language has also been labelled *sensory sound symbolism* (Cuskley & Kirby, 2013), in other words the phenomenon where a word’s form imitates its referent (Lockwood & Dingemanse, 2015). Absolute iconicity is perhaps most prominently exemplified in spoken language by ideophones – vivid sensory words such as the Japanese ‘*pika-pika’* (bright and shiny) or the Siwu ‘*gidigidi’* (running energetically). Unlike English, which incorporates onomatopoeic words into conventional grammatical categories, many languages treat ideophones as a distinct word class with unique syntactic and phonological characteristics.

In relative iconicity, (what Cuskley and Kirby (2013) refer to as *conventional sound symbolism*) the relationships between multiple forms are analogous to relations between different meanings. This can be thought of as a mapping between two or more words and two or more referents, such as phonesthemes like the English ‘str-’, which denotes something long and thin, as in ‘*straight’*, ‘*stripe’*, ‘*stream’*, and ‘*string’* (though it is worth noting that it is debated whether phonesthemes have a direct sensory link to associated meaning, as such ‘pockets of consistency’ in the lexicon may arise simply by chance (Baayen et al., 2011)). However some phonesthemes for example ‘sn-’ (occurring in words relating to the nose – ‘*sniff’*, ‘*snore’*, ‘*sneeze’*) do elicit sensory imagery and could therefore be considered iconic, Schmidtke et al. (2014). The term *conventional sound symbolism* can also cover correlations between sounds and grammatical categories – which is a form of non-arbitrariness called *systematicity* (Dingemanse et al., 2015; Monaghan et al., 2014). A good example is nouns denoting abstract concepts being longer and more derivationally complex than concrete nouns (Reilly & Kean, 2007). This is a form of non-arbitrariness as it reveals connections between word forms and meanings; however it primarily concerns broad statistical patterns between groups of words across the lexicon, rather than the mappings between specific words and their meanings (see Dingemanse et al. (2015) for a detailed review).

This paper will first briefly review evidence for some of the different forms of sound symbolism in human language, and build on existing work (Dingemanse et al., 2015; Sidhu, 2019; Sidhu & Pexman, 2018; Svantesson, 2017) to explore how these form-meaning relationships might arise. We will then turn our attention to the possible role of sound symbolism from an ontogenetic and evolutionary perspective. Finally, we incorporate evidence for iconicity in non-human communication within a neurobiological framework for human language processing to suggest that sound-symbolism, in addition to arbitrariness, should be viewed as integral to language, rather than a marginal linguistic phenomenon.

## Sound Symbolism

Spoken languages consist of an inventory of different sounds, or phonemes, which can be combined in different ways to form words. These words are then attributed to meanings, leading to the fundamental questions about the nature of this form-meaning relationship raised above. Primarily, as a foundation for spoken language, do phonemes themselves convey inherent qualities which lead to association with certain meanings, or are they semantically neutral in nature? The notion that phonemes may carry such associations underlies the phenomenon of sound symbolism. There are different ways in which phonemes may evoke semantic properties, examples of which are presented below.

### Sound-Size Symbolism

**Figure 1.**
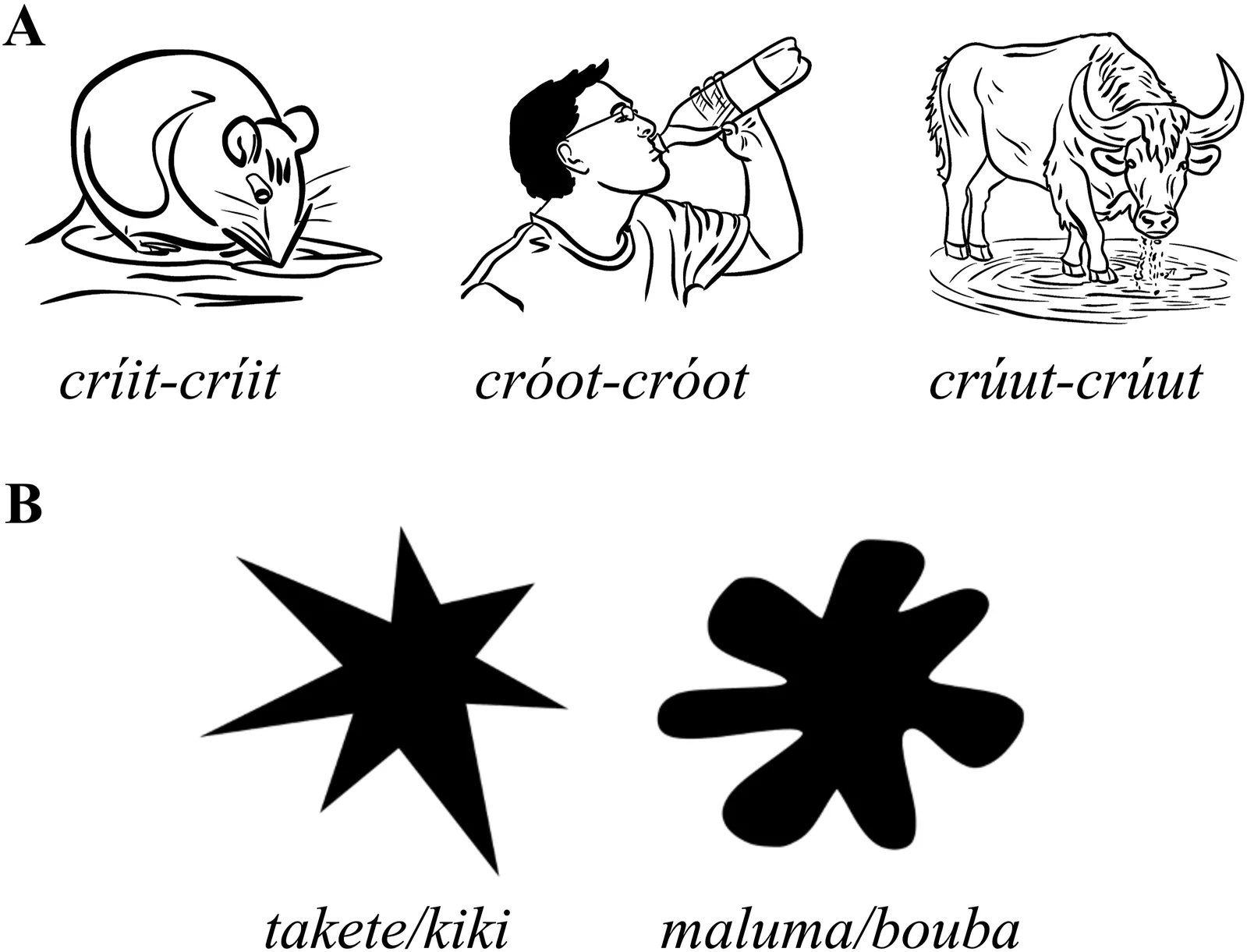
(a) The use of vowel variation to indicate size differences in Khmu ideophones (Svantesson, 2017). (b) The takete/maluma or bouba/kiki effect - when prompted most subjects call the angular shape (left) *takete/kiki* and the rounded (right) shape *maluma/bouba*.

One of the best-known examples of sound symbolism is the idea that different vowels may communicate the semantic property of either small or large size. Almost a century ago it was first reported that a closed-front vowel /i/ may more naturally denote small size over an open-front vowel /a/ (Sapir, 1929). Sapir presented participants with short nonwords such as *‘mil’* and *‘mal’* that were allocated to meanings such as ‘*table’*. Participants were then asked to distinguish these nonwords according to their perceived size and decide which nonword symbolised a larger object. Between 75%–96% of responses were in favour of the nonword containing /a/ as referring to the larger referent, consistently across different ages and language backgrounds (although with a large variance in individual sensitivity to the ‘symbolic suggestiveness’ of phoneme features). This was later extended to include a more gradual scale, as well as vowels in the front-back dimension (ɔ = o > u = a > æ > ε > e > i; Newman, 1933). Outside of forced-choice experiments, the association between different phonemes and the depiction of size has since been corroborated by cross-linguistic evidence, with some languages containing very elaborate size scales. One example is Khmu, the language of the northern Laos region. According to Svantesson (2017), in Khmu ideophones (words that represent sensory imagery), vowel variation is used to indicate size differences, as illustrated by the expressions for ‘drink noisily’ (*crúut-crúut > cróot-cróot > críit-críit > créet-créet)*. The expression chosen may vary according to the size of the drinking animal - for example, *crúut-crúut* could describe the sound of a buffalo drinking noisily, *cróot-cróot* the sound of a human, and *críit-críit/créet-créet* the sound of smaller animals (Figure 1(a)).

There is also evidence to suggest that it is the number of ‘small’ or ‘large’ vowel sounds in a word, and not vowel alone, that determines the conveyed magnitude (Thompson & Estes, 2011). In this study, English-speaking participants were presented with novel figures of varying size and asked to match them to nonwords containing either ‘large’ (*u, o*) or ‘small’ (*i, e*) vowels. The authors found a linear relationship between the size of an object and the number of ‘large’ phonemes in its preferred name, regardless of whether the nonwords were presented aurally or in writing.

### Sound-Shape Symbolism

Another well-documented example of sound symbolism is the *maluma/takete* or *bouba/kiki* effect, which demonstrates a connection between specific phonemes and the perception of shapes like roundness or sharpness. Köhler (1929) was the first to observe that participants assigned non-words *takete* and *maluma* to spiky and rounded shapes, respectively (Figure 1(b)). Decades later, Ramachandran and Hubbard (2001) replaced the terms with *bouba* and *kiki* and reported that 95% of English-speaking adult participants matched *bouba* with the rounded shape and *kiki* with the spiky one. Although they did not provide data to support this prevalence estimate, numerous subsequent studies have confirmed the effect across age groups, including infants and young children (Maurer et al., 2006; Ozturk et al., 2013). A meta-analysis by Fort et al. (2018) found a modest but consistent *bouba/kiki* effect across diverse languages and paradigms, with greater sensitivity for rounded shapes than spiky ones.

While the *bouba/kiki* effect is robust and cross-culturally documented, its relevance to natural spoken language remains debated. Some studies failed to replicate the effect (Rogers & Ross, 1975; Styles & Gawne, 2017) and argue that the effect is determined by whether test words conform to the sound structure of the target language. Sidhu and Pexman (2018) question whether results from forced-choice experiments used to test the *bouba/kiki* effect can be generalised to natural language, noting that experimental settings may heighten participants’ awareness of shared properties between stimuli. Another issue is the potential role of orthography, with some authors suggesting that the *bouba/kiki* effect may be partly driven by the visual shape of written symbols, given that most study participants are literate (Cuskley et al., 2017). For instance, Koriat and Levy (1977) proposed that letters representing rounded sounds (e.g., /u/, /b/) often have curved shapes, while those representing spiky sounds (e.g., /k/, /t/) are angular. However, studies like Bremner et al. (2013), which observed the effect in the non-literate Himba population of Northern Namibia (albeit at a reduced prevalence of 82%), suggest that it is not entirely dependent on orthography. A comprehensive analysis by Ćwiek et al. (2022), spanning 25 languages and 10 writing systems also found no consistent relationship between orthographic shape and the *bouba/kiki* effect, providing strong evidence that the phenomenon arises independently of written language.

Debate also surrounds the question of which phonemes drive the effect. Some authors emphasise the role of vowels, particularly the close back rounded vowel /u/ associated with rounded shapes (Maurer et al., 2006; Ramachandran & Hubbard, 2001), while others highlight the contribution of consonants, noting that the harsh stop consonant /k/ contrasts with the softer bilabial /b/ (Nielsen & Rendall, 2011; Westbury, 2005). Evidence suggests that both play a role. For example, Nielsen and Rendall (2013) demonstrated that sound-shape symbolism is influenced by multiple phoneme categories, and Westbury et al. (2018) linked specific phonemes (e.g., /oƱ/ (as in *soak*), /u/, /b/, /m/ and /ɑ/ for roundness, and /t/, /k/, /z/, /i/, and /ɪ/ (as in *sing*) for spikiness) to subjective shape associations in a large dataset of 8000 randomly generated non-words. Extending this work to real words, Sidhu et al. (2021) found that English nouns describing round objects more frequently contained round-associated phonemes (/u/, /m/, /oƱ/, /b/) than words for spiky objects, which were more likely to include spiky-associated phonemes (/k/, /t/, /l/).

### Other Phoneme-Feature Associations

Although much research on sensory sound symbolism has focused on the *sound-size effect* and *sound-shape effect*, several other phoneme-feature associations have been described in the literature (see Lockwood and Dingemanse (2015) for a detailed review). For instance, Vainio and Vainio (2021) describe the phenomenon of sound-action symbolism, in which vocal sounds are associated with a particular body action. An example is the sound-grip effect, in which a precision grip action is associated with front-close vowels and voiceless stop consonants, and a power grip is associated with low-back vowels. This is a form of sound symbolism in which certain vocal signs have connections to motor, perceptual and conceptual representations of particular hand actions. In another study, Hirata et al. (2011) observed that lightness influences sound sensitivity – participants were more successful at identifying consonants when they experienced congruent sound–light pairings (e.g., voiceless consonants with light visual stimuli and voiced consonants with dark visual stimuli) compared to incongruent pairings. A further example of phoneme-feature association comes from a study which investigated how front and back vowels affect conceptual precision (Maglio et al., 2014). Participants showed greater precision in geographic and action descriptions for labels containing front vowels compared to back vowels. Back vowels in product names however made participants focus on long-term benefits rather than immediate features. This evidence highlights how different vowel sounds can influence mental representation.

Sound symbolic relationships have also been noted for features such as taste (Gallace et al., 2011; Simner et al., 2010), colour (Johansson et al., 2020), and perception of dominant or submissive body postures (Auracher, 2017), suggesting that sound symbolism in language extends far beyond the *takete/maluma* effect described by Köhler. There is even evidence to suggest that people may draw upon knowledge of form-meaning resemblances in open-ended situations (Davis et al., 2019). When asked to ‘draw a creature’ described by nonce words, participants included similar elements in drawings, demonstrating that sound symbolism evokes expected properties of referents. These are only some of the examples of phoneme-feature associations described in the literature. Mechanisms of such associations and their consequences, both behavioural and evolutionary, are explored in subsequent sections.

## Mechanisms of Sound Symbolism

The literature on the mechanisms of sound symbolism largely centres around cross-modal correspondences, defined as ‘a compatibility between attributes or dimensions of a stimulus (i.e., an object or event) in different sensory modalities’ (Spence, 2011, p. 3). The mechanisms of sound symbolism have been comprehensively reviewed by Sidhu and Pexman (2018), who evaluated five proposals, along with their supporting evidence. We summarise these briefly below and discuss additional evidence for each mechanism where appropriate.

According to Sidhu and Pexman (2018) one way that sound-symbolic association may be explained is through *statistical regularities and co-occurrences* between phonetic features and associated stimuli in the environment. This may be understood via Bayesian integration theory (Spence, 2011), whereby humans combine prior knowledge and sensory information to make cross-modal associations about stimuli. For example, pointed objects tend to produce less tonal sounds with more abrupt transitions whereas rounded forms produce more continuous, tonal sounds, and these statistical co-occurrences could be reflected in language (McCormick et al., 2015). Recent computational modelling by Fort and Schwartz (2022) indeed implies that the *bouba/kiki* effect might rely on acoustic cues of spectral balance and temporal continuity. They suggest that cognitively, a sound may be perceived as either spikey or round because it is likely to be produced by a spikey or round object hitting/rolling on a hard surface. Rounded objects, when compared with spiky objects, have lower frequency acoustic resonance modes, meaning that they produce sounds with more energy in the low-frequency part of the acoustic spectrum. They also have smoother trajectories when rolling on a hard surface and therefore produce more continuous acoustic envelopes. These properties, it is argued, may contribute to the formation of cross-modal correspondences responsible for human sensitivity to sound symbolism.

As noted by Sidhu and Pexman (2018), the statistical co-occurrences explanation relies upon experience, and therefore assumes that there is no innate human sensitivity to sound symbolism. Therefore, according to this explanation alone, it might be expected that sound-symbolic effects are not present at birth or in congenitally deaf individuals, yet sound symbolism effects have been observed in 4 month old infants (Ozturk et al., 2013; Peña et al., 2011) and although the effect is diminished in individuals with prelingual auditory deprivation, it is still present at above-chance levels (Gold & Segal, 2020). It is quite possible that statistical learning begins from birth, however it seems plausible that humans may also have an innate sensitivity to sound symbolism. A related view therefore argues that statistical co-occurrences underpin sound symbolism, but that these associations are *innate* and not acquired (Ohala, 1994). According to this account, the association between high frequency and smallness, and low frequency and largeness, is innate and universal across species. It is however difficult to generate testable hypotheses to test this proposal, and a distinction is yet to be made between potential innate statistical associations and cross-modal mappings acquired from birth.

Another potential mechanism that also relies on statistical co-occurrences - though this time occurring within language itself - is what Sidhu and Pexman (2018) label *language patterns*. Here, sound symbolic associations are argued to emerge from co-occurrences between phonological and semantic features, with repeated exposure to phoneme clusters with similar meanings (e.g., phonesthemes) leading to formation of iconic associations. Such patterns can then be used to generate original words, for instance participants using ‘*gl-’* (as in ‘*gleam’*, ‘*glisten’*, ‘*glow’)* to create a novel word related to brightness (Magnus, 2001). However, as noted previously, it is unclear whether such language patterns can be considered to underpin or emerge from sound symbolic associations.

Sound-symbolic associations have also been explained via the broad mechanism of *shared properties between phonemes and associated stimuli*, which Sidhu and Pexman (2018) argue includes both perceptual links between phonemes and articulatory gestures, as well as higher level conceptual links shared across modalities, for example higher pitch having a connotative association with sharpness. As an example of the former, Sapir (1929) hypothesised that participants might associate high front vowels (e.g., /i/ in ‘mil’) with small shapes, as the articulation of such vowels requires a smaller oral cavity. In this way, the shape and phoneme share the property of smallness. Shared properties may also be used to explain the *bouba/kiki* effect, as the pointed lines in the ‘*kiki’* shape mimic sharp phonemic inflections of the sound ‘*kiki’*, as well as sharp inflection of the tongue on the palate (Ramachandran & Hubbard, 2001). Similarly, the round shape may be labelled ‘*bouba’* due to the corresponding roundness of mouth and lips when producing the phoneme /u/. In this way, there is a physical relationship between mouth shape and referent object shape, which may provide grounds for resemblance-based association. Ramachandran and Hubbard (2001) suggested that this may occur via cross-modal cortical connections between representations of mouth shapes in motor areas and phonemic representations in proximal auditory regions of the brain. These sensorimotor mappings are proposed to be similar to those in synaesthesia (where a stimulus in one modality triggers the expected sensory experience, as well as activation in at least one other modality) and facilitate non-arbitrary links between an object’s visual form and its neural representation. The authors argued that the angular gyrus is important for this cross-modal association, as it is well-positioned between temporal, parietal, and occipital lobes. They also provide neuropsychological evidence of a patient with left angular gyrus damage that did not exhibit the *bouba/kiki* effect. Ramachandran and Hubbard’s proposal was questioned by Ikegami and Zlatev (2007), who argued that since shape symbolism is not involuntary and often not consciously perceived, it is fundamentally distinct from synaesthesia. The shared properties mechanism is also explored in the Front Oral Cavity (FOC) theory (Masuda, 2007), which proposes that the front oral cavity resonance frequency (which is high for high front vowels) may be the acoustic basis for an association between sound and meaning, and that kinaesthetic factors, such as tongue movement, may contribute to the effect. The FOC theory encompasses acoustic and articulatory bases, providing a possible explanation as to why deaf subjects are still sensitive to sound symbolism (Eberhardt, 1940) – they may feel the size of the oral cavity, and use other visual factors such as the degree of lip rounding.

## Sound Symbolism in Language Acquisition and Language Evolution

Having provided an overview of the key forms of sound symbolism, and the proposals of their mechanisms discussed in the literature, we now turn to exploring the possible roles of sound symbolism and iconicity in both language acquisition and in the context of language evolution. We also use this evidence to introduce a novel framework for understanding how iconicity might fit into a neurobiological model of human language processing.

### Sound Symbolism in Language Acquisition

The advantages of sound symbolism in language acquisition have been well documented. It has been found that words learned earlier tend to be more iconic, suggesting that sound symbolism may support word learning (Perry et al., 2015). A study by Imai et al. (2008) suggested that iconicity facilitates early verb learning, and experiments in adults have shown that sound symbolism in natural language may aid word learning too (Lockwood et al., 2016). Sound symbolism may facilitate word learning via ‘grounding’, whereby resemblance-based form-meaning relationships allow for shared understanding and the establishment of communication (Cuskley & Sommer, forthcoming). For example, if iconic mappings are shared (e.g., /i/ and smallness), when combined with other multimodal inputs (e.g., pointing) this may facilitate shared attention and generation of a form-meaning relationship. A shared understanding of intuitive form-meaning associations may thus facilitate the formation of a lexicon – in other words sound symbolism may act as a scaffold for mapping semantic information, thus bootstrapping word learning (Imai & Kita, 2014; Monaghan et al., 2014). Thus, sound symbolism may aid bootstrapping via establishing ‘referentiality’ (an ability to map linguistic form to meaning) – a process fundamental to language learning, in combination with Hebbian learning and joint attentional processes (Perniss & Vigliocco, 2014). In a recent review, Nielsen and Dingemanse (2021) found strong evidence for the role of sound symbolism in ‘local learning enhancement’ - where resemblance-based associations of certain lexical items influence the learning of those items - but not for ‘general learning enhancement’, where they influence the later learning of arbitrary items. In addition, it has been suggested that iconicity aids the comprehension of communicative signs (Perniss & Vigliocco, 2014) and that the imitative, performative nature of iconic words makes communication more vivid (Lockwood & Dingemanse, 2015). It has however also been noted that more research is required on these effects of sound symbolism, as there may be cross-linguistic variation in learning benefits.

### Iconicity in Language Evolution

Whilst the role of sound symbolism in language acquisition has been well established, its role in the evolution of language is a matter of continuing debate. The beginning of use of words as a communicative means was arguably a pivotal point in human evolution, with small articulations permitting rapid and efficient encoding of information, as well as wide and distant broadcast and communication without sight. However, given the relatively short period of recorded human history, a cross-species comparison is necessary to answer questions relating to language evolution, and the role of iconicity in this context.

The core platform for language is face-to-face communication, as this is how languages are learnt and most used. Manual gesture is seen as a likely evolutionary precursor to vocal communication (Rizzolatti & Arbib, 1998), and iconicity has been considered as a logical entry into the language system (Armstrong, 1983), perhaps acting as a bridge between manual (gesture) communicative systems and the verbal language we use today. For example, Levinson and Holler (2014) propose an evolutionary stratification of human communicative systems, whereby the different layers (e.g., joint attention, iconic gesture, turn-taking, and vocalisations) vary in antiquity. Declarative pointing is a form of signalling which facilitates mutual gaze to objects and thus allows for joint attention, while iconic gestures allow the effective depiction of motion, size and spatial relations between objects, such as an offering of something presented with the hand or other body parts (Liebal et al., 2006) or a request made with an open hand (Pollick & De Waal, 2007). The repeated use of iconic gestures would have arguably facilitated the grounding and memorisation of representations, leading to conventionalisation and hence increasing levels of abstraction (Garrod et al., 2007). In this way, iconicity may have been important for achieving *displacement*, in other words the ability to refer to things that are spatially and/or temporally remote (Perniss & Vigliocco, 2014). Displacement would be required to allow hominins to progress from a communication system based on functional reference and pointing to a system based on conceptual reference, and iconicity may therefore have contributed to the development of the cognitive ability required to use conceptually referential signals.

While the hypothesis above argues for gesture as a precursor to language in the vocal domain (e.g., Rizzolatti & Arbib, 1998), Perniss et al. (2010) reject this claim, proposing instead that language in manual and vocal modalities must have co-evolved, as linguistic and imagistic components are tightly integrated. This embodied link between linguistic form and sensorimotor experience is argued to reduce cognitive ability needed to unite signs and referents, with linguistic form activating the same systems used in perception and action. The finding that ideophones are more common in narrative contexts and occur alongside iconic gesture (Dingemanse, 2013) is taken as evidence for embodiment in language, with iconicity and gesture contributing to a multimodal act of depiction. According to this account, the innateness of embodiment is shown by close connections between the hand and mouth in the somatotopic organisation of the human motor cortex (Meier et al., 2008) and in congenitally blind individuals who gesture while speaking despite never having observed this (Iverson & Goldin-Meadow, 2001).

There is also some evidence that non-human primates may utilise the processing advantages of embodiment through iconic gestural communication. Studies have demonstrated that orangutans and chimpanzees can use iconic gestures (pantomime) to represent objects and mimic actions related to their use. Notably, these apes often elaborate on gestures that fail to elicit the desired response from their recipient, showcasing flexibility in their communication. This behaviour has been observed in both great apes raised in captivity (e.g., Miles et al., 1996; Tanner et al., 2006), and in forest-living rehabilitant orangutans (Russon & Andrews, 2011). However, the evidence for the use of iconic gestures in apes remains contested. Some researchers argue that non-human primates do not produce truly iconic gestures, as there is no clear requirement for the recipient to infer a resemblance between the gesture and its intended meaning (see Byrne et al., 2017; Tomasello & Call, 2019). Adding nuance to this debate, Perlman et al. (2012) point to a continuity between gesture and instrumental action in apes, suggesting that gestures are influenced by immediate physical and social contexts and can be adapted into spontaneous iconic gestures on-the-spot.

Of course, the great difficulty when examining the relationship between form and meaning from an evolutionary perspective is that it is highly contested whether animal signals can be said to have meaning (i.e., whether non-humans cognitively represent referents; Moore, 2014; Rendall et al., 2009; Scott-Phillips, 2015). This has led some authors to develop alternative cross-species approaches to examining arbitrariness and iconicity, such as Watson et al.’s (2022) ‘optionality’ framework, which highlights the presence of linguistic features of arbitrariness in non-human communication. Others (Fischer & Price, 2017) argue that non-human primates do not express communicative or informative intent, and so non-human communication (vocalisations and manual gesture) should be conceived as goal-directed behaviour only, with nothing more than a probabilistic causal link between the signifier and signified. For example, a chimpanzee may wave an arm in a ‘beckoning’ gesture to another simply because this produces the desired response in the recipient. Warren and Call (2022) however argue that non-human primates may be capable of applying social inferences to a communicative act, and that there may be mentalistic processes which underlie outcomes. Their model of ‘inferential communication’ argues that visual perspective taking and knowledge attribution are required for communicative exchange, helping to bridge the gap between animal and human communication.

Yet another useful approach to analysing the role of iconicity in language evolution is to consider behavioural imitation of others. The human ability to imitate the actions of others is fundamental to turn-taking, which is one of the critical foundations of language evolution (Levinson & Holler, 2014). Imitation is supported by mirror neurons, which code for manual goal-directed movement and fire both when an individual performs a manual task as well as when watching another individual perform the same task. They allow recognition of another’s action, as the same neural activation necessary to produce an action is generated via observation. This is important for the development of mutual understanding and an ability to share meaning. Iconicity and the mirror neuron system may therefore contribute to the emergence of expressions, as is seen in signed languages where new signs emerge from iconic gesture (Ahlner & Zlatev, 2010). In non-human primates, mirror neurons have been found in the ventral premotor cortex – comparable to the location of the mirror neuron system in humans - and are argued to have provided a bridge between iconic gesture and the imitation of behaviour (Rizzolatti & Arbib, 1998; see also Ramachandran & Hubbard, 2001). There is also evidence that chimpanzees and other great apes show imitative abilities beyond those of other animals (Bates & Byrne, 2010), which challenges the long-held view that humans are ‘imitators’ but non-human primates are ‘emulators’ (e.g., Tennie et al., 2006; Tomasello, 1996; Tomasello et al., 1987). It is argued that similarities in cultural transmission across species indicate our common ancestor was capable of imitating actions with sufficient fidelity to transmit culturally variant behaviours within and between communities (Whiten et al., 2009).

Pulling all this evidence together therefore suggests that speech may have evolved from proto-dialogue between individuals based on iconic hand gestures and imitation, supported by mutual action recognition facilitated by the mirror-neuron system (Rizzolatti & Arbib, 1998). While sound-symbolism in contemporary language may be perceived as a vestige of the iconic protolanguage (Kita et al., 2010), its manifold roles and advantages in the context of language evolution arguably imply a more integral role within the human language system.

### Iconicity Within the Neurobiological Framework for Human Language

According to one prominent model, the Dual Neurobiological Systems Hypothesis (DNS, Marslen-Wilson & Tyler, 2007), modern human communicative capabilities are controlled by joint activation of bihemispheric (BH) and left-lateralised neural systems, which interact but are functionally and evolutionarily distinguishable (Marslen-Wilson & Bozic, 2018). Broadly speaking, the left-lateralised system encompasses a network of left-hemisphere frontal and temporal regions surrounding the Sylvian fissure and is unique to humans and responsible for supporting complex syntactic functions. The bihemispheric system involves a broad network of fronto-temporal regions in both hemispheres. It underpins social communication, through the processing of sound-to-meaning mapping, pragmatics, linear adjacency and multimodal interpretation (Bozic et al., 2010, 2015; Marslen-Wilson & Bozic, 2018) – with its capacity for interpretation of multimodal social communication cues particularly relevant for the current context. The bihemispheric system is argued to be evolutionarily primary, with the evidence showing that this system and its functions are highly conserved in non-human primates (Ghazanfar et al., 2008; Seyfarth & Cheney, 2017; Wilson et al., 2015). As such, the bihemispheric system can be considered a promising analogue to the one present in humans at the early stages of language evolution. Given that most forms of iconicity are underpinned by ‘linguistic cross-modal correspondence’ (Cuskley & Sommer, forthcoming; Sidhu & Pexman, 2018) - excluding only the most direct form-meaning associations such as onomatopoeia that remain within one sensory modality - and the presence of cross-modal associations has also been noted in non-human primates (Ludwig et al., 2011), it is possible that iconicity may have played a role in bridging the gap between the gestural or imitative visual signs used in early communication and the lexicalised concepts that emerged later. According to this view, cross-modal transfer may have developed into a more sophisticated cross-modal cognitive suite that provided humans with the neurological and behavioural architecture capable of storing and increasing cross-modal representations, thus affording us the ability to learn arbitrary symbols necessary for the modern linguistic capacity (Cuskley & Sommer, forthcoming). Therefore, iconicity may in part explain the evolutionary gap between the primate and the modern bihemispheric systems, ultimately setting a precedent for advancing communicative abilities that led to the evolution of the human linguistic capability – with the bihemispheric system likely heavily involved in the processing of iconic sounds, in line with evidence for activation in the bilateral superior temporal sulci (STS) in response to sound symbolic words (Kanero et al., 2014). Taking this evidence into account, we argue that placing iconicity within the neurobiological framework of the DNS hypothesis, and the bihemispheric system in particular, could help strengthen the argument that iconicity played a prominent role in early human language evolution.

## Factors Limiting the Prevalence of Iconicity

If iconicity provides so many advantages, and is believed to be a stepping stone in the evolution of language, why is language still predominantly arbitrary? From an evolutionary perspective, features of language that improve processing and learnability should survive and become more common. Indeed, Jesperson (1922) found evidence of /i/ replacing other vowels in the historical development of words with meanings related to small size. This may have occurred as sound symbolic associations of /i/ made words containing this phoneme and describing small objects more favourable, and more likely to survive in language evolution – leading to the claim that languages should become ‘richer and richer in symbolic words’.

There are however factors that limit the type of meanings that can be expressed iconically. Dingemanse et al. (2015) and Sidhu (2019) review several such factors. Firstly, iconic words must have a fairly unique meaning, as similar meanings beg similar forms, and so iconicity may lead to ambiguity unless the referent is very distinct. A wholly iconic language would be dominated by words with similar forms and meanings, leading to uncertainties and deficiencies in processing and learnability. Secondly, the amount of sensory information contained within a referent may limit whether or not it can be described via an iconic relationship. Iconic mappings require sensory features, which restricts their use in language systems. Lupyan and Winter (2018) expand on this further, arguing that iconicity is limited in the expression of abstract concepts in particular, as iconic expressions are too strongly linked to sensory features or certain contexts. For example, certain metaphorical extensions found in English and Hebrew are not possible in Israeli Sign Language (ISL) and American Sign Language as the signs are too iconic (Meir, 2010). One example given is that while English allows for the verb “eat” to extend metaphorically (e.g., “The acid ate the iron key”), ISL’s sign for “eat” is too specific, depicting a human eating action at the mouth, which restricts such extensions. Meir discusses how this limitation is also seen in spoken languages where iconic sound-related words are less likely to be used metaphorically across sensory dimensions in ‘synaesthetic metaphors’. These examples demonstrate how iconicity may limit the meaning of an expression to a particular context, and thus its potential to become more abstract.

Iconicity in language may also be influenced by linguistic laws such as Zipf’s law of abbreviation, which predicts a negative relationship between word length and frequency of use (i.e., words used more frequently tend to be shorter and vice versa). Zipf’s law, alongside Menzerath’s law (which states that longer communicative constructs are composed of shorter parts) captures the information theoretic principle of minimising code length (compression). Both laws are ubiquitous in communicative systems, having been observed in both manual gesture and vocal communications of non-human primates as well as humans (Heesen et al., 2019; Huang et al., 2020). These laws may decrease iconicity in language - as frequency of use increases, the need for efficiency prevails over iconicity, resulting in ‘lexical elaboration’ with increasing levels of arbitrariness (Haiman, 1985).

Iconic words, such as ideophones, also seem to resist deep integration into the grammatical apparatus of a language. According to Dingemanse (2017), this reflects conflict between grammatical integration and the strength of iconic form-meaning mappings – if fully integrated, iconic expressions are subjected to processes of reduction and regularisation, so iconicity is reduced. This syntactic conventionalisation diminishes the role of iconicity and could perhaps be further evidence for the processing of iconic signals within the bihemispheric system, as it is the specialised left-hemisphere frontotemporal system that provides the principal neurobiological substrate of core, distinctly human-specific, grammatical capacity, (Bozic et al., 2010, 2015) and iconicity seems to resist grammatical integration.

The prevalence of iconicity in language is also limited by the advantages of arbitrariness. According to (Hockett, 1963), the flexibility of signalling afforded by arbitrariness is a fundamental property of language. Arbitrariness allows referral to any possible concept, and unlike iconicity it can allow for easy discrimination between similar entries in a lexicon. This allows for larger lexica to develop (Gasser, 2004) and allows communication about concepts for which direct perceptual grounding is not possible. A highly iconic lexicon with similar phonological forms for similar meanings would lead to high confusability, and decreased communicative effectiveness (Perniss et al., 2010).

## Conclusions

In summary, the ancient debate over whether the linguistic sign is arbitrary has been clouded by unnecessary polarisation. A resemblance between form and meaning cannot be sufficient for understanding meaning, and conventionalisation of language throughout evolution is inevitable and necessary to maintain the efficiency and versatility of language. Hence while much of the historic debate until recent years has been binary, words cannot be entirely arbitrary or iconic – they fall on a spectrum instead. Iconicity and arbitrariness both convey their own unique advantages in a linguistic system, representing the adaptation of languages to the constraints of needing to link linguistic form with human experience whilst ensuring an effective signal. It is these constraints that drive the evolution and development of linguistic systems, adding further weight to the hypothesis that theories of language (both phylogenetic and ontogenetic) must value iconicity in addition to arbitrariness, and sound symbolism should be regarded as a core principle of language, and not merely a peripheral phenomenon.

## Data Availability

Data sharing not applicable to this article as no datasets were generated or analysed during the current study.
